# Neutrophils Facilitate Prolonged Inflammasome Response in the DAMP-Rich Inflammatory Milieu

**DOI:** 10.3389/fimmu.2021.746032

**Published:** 2021-09-29

**Authors:** Seunghwan Son, Sung-Hyun Yoon, Byeong Jun Chae, Inhwa Hwang, Do-Wan Shim, Young Ho Choe, Young-Min Hyun, Je-Wook Yu

**Affiliations:** ^1^ Department of Microbiology and Immunology, Institute for Immunology and Immunological Diseases, Brain Korea 21 Project for Medical Science, Yonsei University College of Medicine, Seoul, South Korea; ^2^ Department of Anatomy, Brain Korea 21 Project for Medical Science, Yonsei University College of Medicine, Seoul, South Korea

**Keywords:** neutrophil, inflammasome, pyroptosis, DAMP, NLRP3 desensitization, SARM1, efferocytosis

## Abstract

Aberrant inflammasome activation contributes to various chronic inflammatory diseases; however, pyroptosis of inflammasome-active cells promptly terminates local inflammasome response. Molecular mechanisms underlying prolonged inflammasome signaling thus require further elucidation. Here, we report that neutrophil-specific resistance to pyroptosis and NLRP3 desensitization can facilitate sustained inflammasome response and interleukin-1β secretion. Unlike macrophages, inflammasome-activated neutrophils did not undergo pyroptosis, indicated by using *in vitro* cell-based assay and *in vivo* mouse model. Intriguingly, danger-associated molecular patterns (DAMP)-rich milieu in the inflammatory region significantly abrogated NLRP3-activating potential of macrophages, but not of neutrophils. This macrophage-specific NLRP3 desensitization was associated with DAMP-induced mitochondrial depolarization that was not observed in neutrophils due to a lack of SARM1 expression. Indeed, valinomycin-induced compulsory mitochondrial depolarization in neutrophils restored inflammasome-dependent cell death and ATP-induced NLRP3 desensitization in neutrophils. Alongside prolonged inflammasome-activating potential, neutrophils predominantly secreted interleukin-1β rather than other proinflammatory cytokines upon NLRP3 stimulation. Furthermore, inflammasome-activated neutrophils did not trigger efferocytosis-mediated M2 macrophage polarization essential for the initiation of inflammation resolution. Taken together, our results indicate that neutrophils can prolong inflammasome response *via* mitochondria-dependent resistance to NLRP3 desensitization and function as major interleukin-1β-secreting cells in DAMP-rich inflammatory region.

## Introduction

Neutrophils are the first and most abundant cells that infiltrate inflamed sites following microbial invasion or tissue injury (1). Upon microbial infection, neutrophils play a key role in host defense by phagocytosing microbes and releasing granule-contained antimicrobial factors and neutrophil extracellular traps (1, 2). Although neutrophils have relatively short lifespan, their sustained antimicrobial activity is detrimental to host cells or tissues (3). During the progression of inflammation, inflammatory cells such as neutrophils infiltrate into the inflamed region, where high levels of damage-associated molecular patterns (DAMPs) or danger signals from injured cells are present. Therefore, the effects of DAMP- or danger signals-rich milieu on the inflammatory potential of infiltrating cells warrant careful investigation.

Of interest, neutrophils also contribute to the resolution of inflammation. The phagocytosis of apoptotic neutrophils by resident macrophages, known as efferocytosis, triggers anti-inflammatory signaling by macrophages to resolve inflammation (4). Defective efferocytosis is a primary cause of prolonged inflammation and subsequent inflammatory diseases (5, 6). Since abnormal neutrophil activity is thought to play an important role in the pathogenesis of chronic inflammatory disorders (3), such as rheumatoid arthritis, neutrophil function must be optimally regulated to avoid unnecessary collateral damage to the host.

The inflammasome is a key molecular complex, mainly present in myeloid cells, to initiate inflammatory responses by inducing inflammatory mediator secretion, like interleukin (IL)-1β or IL-18 (7). In response to microbe- or tissue injury-derived factors, inflammasome sensor molecules, such as NOD-like receptor family, pyrin domain-containing 3 (NLRP3), are converted into their active form and assemble adaptor molecule apoptosis-associated speck-like protein containing a caspase recruitment domain (ASC) and procaspase-1 to form the inflammasome complex (8). The assembled inflammasome promptly leads to caspase-1 activation, which then induces pro-IL-1β and gasdermin D (GSDMD) cleavage into their active forms (9).

Unlike other inflammasome sensor molecules, NLRP3 can be exclusively activated by a broad spectrum of stimuli, ranging from microbial toxins to endogenous metabolites (8). These results suggest that NLRP3 can act as a typical sensor to initiate inflammation in response to diverse abnormalities. Recent studies have closely implicated NLRP3 inflammasome activation in the pathogenesis of many chronic metabolic or degenerative diseases, such as type 2 diabetes, atherosclerosis and Alzheimer’s disease (10–12). Despite the important role of NLRP3 inflammasome in a wide range of chronic diseases, inflammasome activation promptly results in the pyroptotic cell death of inflammasome-active cells by causing the formation of GSDMD pores (13). Consequently, inflammasome-activated cells such as macrophages are unlikely to survive and support sustained inflammasome response. As such, it remains largely unknown which type of stimuli or cells contribute to prolonged inflammasome signaling under physiological conditions that lead to chronic inflammatory diseases.

Intriguingly, recent studies demonstrated that neutrophils do not undergo pyroptosis upon canonical inflammasome activation (14, 15). Moreover, considering the importance of neutrophils in resolving inflammation, it is highly possible to speculate that neutrophils may act as an essential regulator of persistent or sustained inflammation. In this context, the cellular function and physiological significance of inflammasome-active neutrophils requires further clarification. Here, we explored whether the key inflammatory cells macrophages or neutrophils can support sustained inflammation with a prolonged inflammasome response under diverse conditions.

## Materials and Methods

### Mice

C57BL/6 (Orient Bio, Gyeonggi-do, Korea), *Nlrp3* ^-/-^ (Jackson Laboratory, Bar Harbor, ME, USA), *Gsdmd* ^-/-^ (Jackson Laboratory) and *LysM* ^gfp/+^ (provided by Dr. Pilhan Kim, Korea Advanced Institute of Science and Technology) (16) mice were bred at Yonsei University College of Medicine. All mice (C57BL/6 background) were maintained under specific pathogen-free conditions, and female mice were used for experiments at 8–10 weeks of age. Protocols for the animal experiments were approved by the Institutional Ethical Committee, Yonsei University College of Medicine. All experiments were performed in accordance with the approved guidelines of the Institutional Ethical Committee.

### Mice Treatment

Mice were injected intraperitoneally with 1 mg/kg of LPS for 24 h. Peritoneal lavage fluid was obtained by washing twice with 5 mL of PBS and centrifuging (300 × *g* for 5 min) to pellet cells. The collected cells were stained with anti-CD11b, anti-Ly6G, and anti-F4/80 antibodies conjugated with an appropriate fluorescent dye (eBioscience, San Diego, CA, USA). Cell fluorescence was monitored and analyzed using flow cytometry (FACSVerse, BD, Franklin Lakes, NJ, USA). All flow cytometry data are representative of at least three independent experiments.

### Cell Culture

Bone marrow cells were isolated from mouse femurs and differentiated into BMDMs in L929-conditioned medium, as described previously (17). All BMDMs were maintained in L929-conditioned DMEM supplemented with 10% fetal bovine serum (FBS, Gibco, Waltham, MA, USA) and antibiotics. BMNs were obtained from mouse bone marrow cells using an EasySep™ mouse neutrophil enrichment kit (STEMCELL Technologies, Vancouver, Canada) according to the manufacturer’s protocols or using Ficoll-Paque density gradient medium. BMNs were cultured in RPMI 1640 supplemented with 10% FBS and antibiotics and used for experiments. Isolated BMNs were analyzed using flow cytometry after co-staining with anti-CD11b, anti-Ly6G, and anti-F4/80 antibodies (eBioscience). Spleen neutrophils were prepared from mouse spleen-derived single-cell suspension using an EasySep™ mouse neutrophil enrichment kit. Mouse peritoneal macrophages were obtained from the peritoneal lavage fluid five days after intraperitoneal injection with 3% thioglycolate medium (1.5 mL). After removing non-adherent cells, peritoneal macrophages were cultured in RPMI 1640 supplemented with 10% FBS and antibiotics. Immortalized NLRP3-GFP-expressing BMDMs were provided by Dr. E.S. Alnemri (Thomas Jefferson University, Philadelphia, PA, USA). To transfect Sarm1 into BMNs, DOTAP or Lipofectamine 2000 liposomal transfection reagent were used as described previously (18). Briefly, cells were incubated with a mixture of cDNA construct and liposomal reagent in Opti-MEM for 4 h. Then, normal FBS-containing culture medium was added and cells were further incubated for additional 18 h.

### Preparation of Cell-Free Supernatants From Injured Cells

To isolate DAMP-rich conditioned medium, mouse BMDMs were treated with staurosporine (2 μg/mL) for 3 h, washed with fresh medium and incubated for an additional 18 h before the supernatant was collected. Alternatively, mouse BMDMs were subjected to four freeze/thaw cycles and the culture supernatant was collected.

### Reagents and Antibodies

LPS, ATP, nigericin, poly dA:dT, staurosporine, cytochalasin D, valinomycin, and apyrase were purchased from Sigma-Aldrich (St. Louis, MO, USA). JC-1 and MitoSox were obtained from Invitrogen (San Diego, CA, USA). zVAD-FMK,and ac-YVAD-CMK were purchased from Bachem (Torrance, CA, USA). Mammalian expression constructs for mouse Sarm1 (pGW1-Myc-Sarm1) was purchased from Addgene (Watertown, MA, USA). The following antibodies were used for detecting mouse caspase-1 (Adipogen, San Diego, CA, USA), NLRP3 (Adipogen), IL-1β (R&D Systems, Minneapolis, MN, USA), ASC (Santa Cruz Biotechnology, Dallas, TX, USA), β-actin (Santa Cruz Biotechnology), caspase-3 (Cell Signaling, Beverly, MA, USA), gasdermin D (Abcam, Cambridge, MA, USA), and Sarm1 (Cell Signaling).

### Immunoblot Analysis

Cells were lysed in buffer containing 25 mM Tris-Cl (pH 7.5), 150 mM NaCl, 1% NP-40, 1% sodium deoxycholate, 0.1% SDS, and protease inhibitors. Soluble lysates were fractionated using SDS-polyacrylamide gel electrophoresis and transferred to polyvinylidene difluoride membranes. Some supernatants were precipitated using a methanol/chloroform mixture as described previously (19) and immunoblotted. All blot images are representative of at least three independent experiments and have been cropped for presentation.

### mRNA Quantification

Total RNA was isolated using an RNeasy Mini Kit (Intron, Gyeonggi-do, Korea) or TRIzol reagent (Invitrogen, Waltham, MA, USA) and reverse-transcribed using a Power cDNA Synthesis Kit (Intron). Quantitative real-time PCR was performed using SYBR Premix Ex Taq (Takara, Tokyo, Japan) while RT-PCR was performed using AccuPower HotStart PCR premix (Bioneer, Daejeon, Korea). The following primers (mouse) were used: *Il-10*, 5′-CCA AGC CTT ATC GGA AAT GA-3′ and 5′-TTT TCA CAG GGG AGA AAT CG-3′; *Il-6*, 5’-AGT TGC CTT CTT GGG ACT GA-3’ and 5’-TCC ACG ATT TCC CAG AGA AC-3’; *Tnfα*, 5’-CGT CAG CCG ATT TGC TAT CT-3’ and 5’-CGG ACT CCG CAA AGT CTA AG-3’; *Gapdh*, 5’-AAC TTT GGC ATT GTG GAA GG-3’ and 5’-ACA CAT TGG GGG TAG GAA CA-3’; *Sarm1*, 5’-CGC TGC CCT GTA CTG GAG G-3’ and 5’-CTT CAG GAG GCT GGC CAG CT-3’; *β*-*actin*, 5’-CCT TCC TGG GCA TGG AGT CCT G-3’ and 5’-GGA GCA ATG ATC TTG ATC TTC-3’.

### Inflammasome/Caspase-1 Activation Assay

To induce conventional NLRP3 inflammasome activation, cells were primed with LPS (0.25 μg/mL) for 2.5 h and then treated with ATP (2.5 mM) for 30 min. In some experiments, cells were primed with LPS, washed with PBS, and then treated with ATP. To stimulate NLRC4 and AIM2 inflammasome signaling, cells were transfected with flagellin (NLRC4) using N-(2,3-dioleoyloxy-1-propyl) trimethylammonium methyl sulfate (DOTAP) or poly dA:dT (AIM2) using Lipofectamine 2000. Inflammasome activation was determined by the presence of active caspase-1 p20 and active IL-1β in culture supernatant immunoblots and extracellular IL-1β quantification using ELISA. To detect ASC oligomerization, disuccinimidyl suberate (DSS, Thermo Scientific)-mediated cross-linking assays were performed as described previously (20). To determine NLRP3 oligomerization, speck-like aggregates of NLRP3-GFP were assessed by confocal microscopy in NLRP3-GFP-expressing BMDMs.

### Cytokine Production Assay

IL-1β, IL-6, and MMP9 levels in the culture supernatants were quantified using mouse IL-1β, IL-6, or MMP9 ELISA kits (R&D Systems), respectively. Inflammatory cytokines in the culture supernatants were quantified using a Cytometric Bead Array Mouse Inflammatory Kit (BD). All assays were performed according to the manufacturer’s protocols.

### Cell Death Assay

To quantify pyroptotic cell death, extracellular lactate dehydrogenase (LDH) release was measured using a CytoTox96 non-radioactive cytotoxicity assay kit (Promega) and calculated as [extracellular LDH/(intracellular LDH + extracellular LDH) × 100]. Dead cells were labeled with PI or Annexin V-FITC according to the manufacturer’s protocol and fluorescence was measured using flow cytometry (FACSVerse, BD). All flow cytometry data are representative of at least three independent experiments. To assess efferocytosis, peritoneal macrophages were treated with untreated, apoptotic, or inflammasome-activated neutrophils (1/4 volume) for 1 h, washed to remove non-engulfed cells, and treated with LPS (0.1 μg/mL) for 18 h.

### Immunofluorescence Assay

Cells grown on coverslips in a 12-well plate were fixed with 4% formaldehyde and permeabilized with 0.2% Triton X-100. After blocking with 4% BSA, cells were incubated with anti-α-tubulin primary antibodies (Santa Cruz Biotechnology) followed by Alexa Fluor 488-conjugated anti-mouse IgG (Invitrogen). F-actin was stained using rhodamine-phalloidin (Thermo) and nuclei were visualized by counterstaining with DAPI. Images were acquired using confocal microscopy (LSM 700, Carl Zeiss, Oberkochen, Germany) and processed using ZEN2011 software.

### Phagocytosis Assay

To measure phagocytic activity, cells were incubated with zymosan-FITC (Invitrogen; 1:5 ratio) for 30 min, washed, and their fluorescence analyzed using flow cytometry (FACSVerse, BD). All flow cytometry data are representative of at least three independent experiments.

### Neutrophil *In Vitro* Migration Assay

To track neutrophil migration, neutrophils were prepared from LysM^gfp/+^ mice. Prior to neutrophil seeding, confocal dishes were coated with fibronectin (Gibco) diluted in PBS. Then, neutrophils were plated with or without LPS (0.25 μg/mL), incubated for 2 h 30 min, followed by the treatment of ATP (2.5 mM) for 30 min or 1 h. *In vitro* migration was imaged using a Nikon Eclipse Ti2 inverted microscope, with DIC and FITC channels captured every 10 s and terminated after 30 min. All imaging data (.nd2) were imported into volocity software v6.3.1 (Perkin Elmer) and processed using IMARIS software v7.2.3 (Bitplane). Tracking analysis was performed using an autoregressive motion in spots tool.

### Mitochondrial Membrane Potential and ROS Measurement

To measure mitochondrial membrane potential, cells were stained with JC-1 (Invitrogen) according to the manufacturer’s protocol and their fluorescence was monitored and analyzed using flow cytometry (FACSVerse, BD). To measure mitochondrial ROS production, cells were stained with MitoSOX (Invitrogen) after the appropriate treatments and analyzed using flow cytometry, based on the level of MitoSOX. All flow cytometry data are representative of at least three independent experiments.

### Statistical Analysis

All values are expressed as the mean ± SEM of individual samples. *n* indicates the number of independent experiments. Data were analyzed using one-way analysis of variance (ANOVA) followed by Dunnett’s *post hoc* test to compare all groups with the control group, or Student’s *t*-test. Statistical significance was set at *p* ≤ 0.05. All analyses were performed using GraphPad Prism.

## Results

### Inflammasome Activation Induces Pyroptotic Cell Death in Macrophages but Not Neutrophils

**Figure 1:**
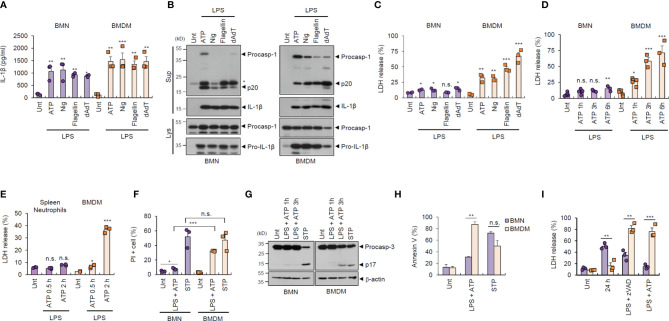
Neutrophils resist inflammasome-dependent cell death. **(A)** Quantification of IL-1β in the culture supernatants of mouse bone marrow-derived neutrophils (BMNs) or macrophages (BMDMs) untreated (Unt) or primed with LPS (0.25 μg/mL, 2.5 h), followed by ATP (2.5 mM, 1 h) or nigericin (Nig, 5 μM, 1 h) treatment, or flagellin (500 μg/mL, 6 h) or poly dA:dT (1 μg/mL, 6 h) transfection (*n* = 3). **(B)** Immunoblots of mouse BMNs or BMDMs treated as in **(A)**. Culture supernatants (Sup) or cell lysates (Lys) immunoblotted with the indicated antibodies. The asterisk indicates a nonspecific band. **(C, D)** Quantification of LDH in the culture supernatants of mouse BMNs or BMDMs treated as in **(A)** (C, *n* = 3) or primed with LPS (0.25 μg/mL, 2.5 h) and treated with ATP (2.5 mM, 1, 3, 6 h) (D, *n* = 3-5). **(E)** Quantification of LDH in the culture supernatant of mouse splenic neutrophils or BMDMs primed with LPS, followed by ATP treatment (0.5 or 2 h). (*n* = 3) **(F)** Quantification of PI-positive mouse BMNs or BMDMs primed with LPS, followed by ATP (2.5 mM, 1 h) or treated with staurosporine (STP, 2 μg/mL, 6 h), as determined by flow cytometric analysis after propidium iodide (PI) staining. (*n* = 3). **(G)** Immunoblots of cell lysates from mouse BMNs or BMDMs primed with LPS followed by ATP (1 or 3 h), or staurosporine (STP, 2 μg/mL, 6 h) treatment. **(H)** Quantification of phosphatidylserine-positive mouse BMNs or BMDMs treated with LPS (0.25 μg/mL), followed by ATP (2 h) or staurosporine (STP, 5 h), as determined by flow cytometric analysis after Annexin V-FITC staining (*n* = 2 or 3). **(I)** Quantification of LDH release into the culture supernatant of mouse BMNs or BMDMs incubated with culture medium (24 h), treated with LPS (0.25 μg/mL, 24 h) in the presence of zVAD (20 μM) or treated with LPS (0.25 μg/mL, 2.5 h) followed by ATP (2.5 mM, 3 h) (*n* = 3). Data are expressed as the mean ± SEM. *P < 0.05, **P < 0.01, ***P < 0.001, n.s. not significant.

To examine the potential outcome of inflammasome activation in major inflammatory cells, we prepared and analyzed bone marrow-derived neutrophils (BMNs) and bone marrow-derived macrophages (BMDMs) from isolated mouse bone marrow cells (**Supplementary Figure S1**). First, we examined the inflammasome response of both cell types following diverse stimulations. NLRP3 inflammasome activation by lipopolysaccharide (LPS) plus ATP or nigericin treatment was observed in BMNs and BMDMs, as determined by extracellular IL-1β secretion (**Figure 1A**). Similarly, neutrophils exhibited normal NLR family, CARD-containing 4 (NLRC4)- and absent in melanoma 2 (AIM2)-mediated inflammasome activation triggered by flagellin or poly dA:dT transfection (**Figure 1A**). Consistently, all forms of inflammasome-activating stimulation caused a robust caspase-1 processing in both cells, as determined by the presence of cleaved caspase-1 (p20) in the cell culture supernatants (**Figure 1B**).

Inflammasome/caspase-1 activation resulted in profound pyroptosis in macrophages, as indicated by lactic acid dehydrogenase (LDH) release into the extracellular medium (**Figure 1C**). Interestingly, this inflammasome-dependent LDH release was not evident in BMNs, irrespective of robust inflammasome activation (**Figures 1C, D**). Consistently, LPS/ATP-induced pyroptosis was not observed in splenic neutrophils (**Figure 1E**). This pyroptosis resistance of neutrophils was also confirmed by using propidium iodide (PI) staining (**Figure 1F** and **Supplementary Figure S2**). However, the well-known apoptosis inducer, staurosporine, promoted robust cell death in neutrophils, as detected by using PI staining and caspase-3 cleavage (**Figures 1F, G**), indicating that neutrophils are susceptible to apoptosis but not pyroptosis. Both neutrophils and macrophages displayed comparable phosphatidylserine exposure levels, a typical eat-me signal, in response to staurosporine, as measured using annexin V staining (**Figure 1H**). However, neutrophils exhibited significantly lower LPS/ATP-induced phosphatidylserine exposure (**Figure 1H**) and cell death (**Figure 1I**) than macrophages. Together, these data demonstrate that neutrophils are inflammasome-competent and secret robust amounts of mature IL-1β, but are resistant to inflammasome-driven pyroptotic cell death.

### DAMP-Rich Milieu Desensitizes the NLRP3 Inflammasome-Activating Potential of Macrophages but Not Neutrophils

**Figure 2:**
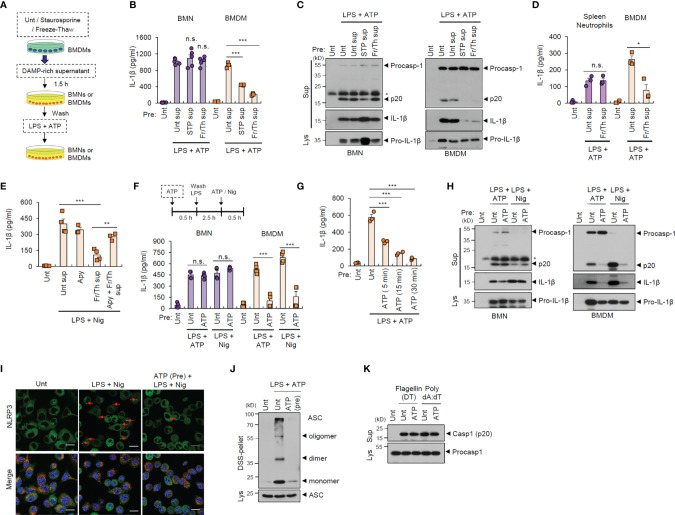
Danger signal-rich medium pretreatment desensitizes NLRP3 inflammasome-activating potential of macrophages but not neutrophils. **(A)** Experimental scheme for determining the potential effect of cell-free supernatants from normal BMDMs or those injured by staurosporine (STP) or repeated freeze/thaw cycles on NLRP3 inflammasome activation of neutrophils or macrophages. **(B)** Quantification of IL-1β in the culture supernatants of mouse BMNs or BMDMs pretreated with cell-free supernatants from untreated or injured BMDMs as in **(A)** and then treated with LPS (0.25 μg/mL, 2.5 h) followed by ATP (2.5 mM, 30 min) treatment (*n* = 5, BMNs; *n* = 3, BMDMs). **(C)** Immunoblots from mouse BMNs or BMDMs treated as in **(B)**. The asterisk indicates a nonspecific band. **(D)** Quantification of IL-1β in the culture supernatants of mouse splenic neutrophils or BMDMs pretreated with cell-free supernatants from untreated or injured BMDMsa by freeze/thaw cycles (2.5 mM, 30 min), and then treated with LPS, followed by ATP (2.5 mM, 30 min) treatment (*n* = 4). **(E)** Quantification of IL-1β in the culture supernatants of mouse BMDMs pretreated with DAMP-rich medium by freeze/thaw cycles in the presence of apyrase (15 U/ml), and then treated with LPS followed by nigericin (5 μM, 40 min) treatment (*n* = 3 or 4) **(F)** Quantification of IL-1β in the culture supernatants of mouse BMNs or BMDMs pretreated with ATP (2.5 mM, 30 min), washed and treated with LPS (0.25 μg/mL, 2.5 h), followed by ATP (2.5 mM, 30 min) or nigericin (Nig, 5 μM, 40 min) treatment (*n* = 4). **(G)** Quantification of IL-1β in the culture supernatants of mouse BMDMs pretreated with ATP (5, 15, 30 min), and then treated with LPS, followed by ATP treatment (*n* = 3). **(H)** Immunoblots from mouse BMNs or BMDMs treated as in **(F)**. **(I)** Representative immunofluorescence images of NLRP3-GFP-expressing immortalized BMDMs pretreated with ATP (2 mM, 30 min) and then treated with LPS (0.25 μg/mL, 3 h), followed by nigericin (Nig, 5 μM, 30 min) treatment after anti-Tom20 antibody staining (red). Nuclei were stained with DAPI (blue). Scale bars, 20 μm. Red arrows indicate NLRP3-oligomerized specks. **(J)** Immunoblots of DSS-crosslinked pellets (DSS-pel) or cellular lysates (Lys) from BMDMs pretreated with ATP (2.5 mM, 30 min) and then treated with LPS (0.25 μg/mL) and ATP (2.5 mM). **(K)** Immunoblots of mouse BMDMs pretreated with ATP (2.5 mM, 30 min) and then transfected with flagellin (500 ng/mL, 4 h) or poly dA:dT (2 μg/mL, 4 h). Culture supernatants (Sup) or cell lysates (Lys) were immunoblotted with the indicated antibodies. *P < 0.05, **P < 0.01, ***P < 0.001, n.s. not significant.

During the initial stages of inflammation, host tissues or cells at inflamed sites are damaged, leading to the extracellular release of many cellular components (21). Consequently, infiltrated cells such as neutrophils are likely to encounter danger signals or DAMPs released by injured cells in the inflamed region (22). To examine whether exposure to danger signal-rich milieu affects the inflammasome-activating potential of infiltrated cells, we exposed neutrophils or macrophages to injured cell-derived supernatants before inflammasome-activating stimulants (**Figure 2A**). *In vitro* cell injury was induced in BMDMs by staurosporine treatment or repeated freeze-thaw cycles. Of notice, pretreatment with DAMP-rich medium did not affect LPS/ATP-induced IL-1β secretion from neutrophils, but significantly decreased IL-1β secretion from BMDMs (**Figure 2B**). Consistently, the exposure with danger signal-rich medium abolished caspase-1 processing in macrophages following LPS/ATP stimulation, but not in neutrophils (**Figure 2C**). These results indicate that danger signals-rich milieu can abrogate macrophage inflammasome-activating potential at the post-translational level. However, similar to BMNs, splenic neutrophils were resistant to DAMP-rich medium-induced NLRP3 desensitization (**Figure 2D**).

Extracellular ATP is a major danger signal released by damaged cells and plays an important role in the inflammaroty processes (23). To examine whether ATP is critical for the DAMP-induced NLRP3 inflammasome desensitization observed in macrophages, we used apyrase to remove ATP in the DAMP-rich medium. Intriguingly, apyrase significantly restored NLRP3 desensitization by freeze-thaw-induced DAMP-rich medium (**Figure 2E**). Then, we pretreated neutrophils or macrophages with ATP before conventional NLRP3-activating stimulation. Similar to the effects observed following treatment with DAMP-rich medium, ATP pretreatment markedly attenuated IL-1β secretion from macrophages, but not from neutrophils, in response to LPS/ATP or LPS/nigericin stimulation (**Figure 2F**). Pre-exposure with ATP for 15 min was sufficient to desensitize LPS/ATP-stimulated inflammasome activation in macrophages (**Figure 2G**). Like DAMP-rich medium, ATP pretreatment markedly blocked NLRP3-mediated caspase-1 cleavage in macrophages, but not neutrophils (**Figure 2H**). We also found that DAMP-rich medium or ATP treatment alone did not trigger a profound production of proinflammatory cytokine in both cells (**Supplementary Figure S3**).

NLRP3 oligomerization is an essential process in the assembly of the NLRP3 inflammasome complex (24). Consistent with the above data, ATP pretreatment remarkably blocked LPS/nigericin-induced NLRP3 oligomerization in macrophages, as determined by NLRP3 speck formation (**Figure 2I** and **Supplementary Figure S4**). Furthermore, ATP pretreatment abolished ASC oligomerization in BMDMs stimulated with LPS/ATP (**Figure 2J**), but did not inhibit NLRC4- and AIM2-dependent caspase-1 processing in macrophages following flagellin and poly dA:dT transfection, respectively (**Figure 2K**). These data demonstrate that extracellular DAMP- or ATP-rich milieu at the inflamed site can desensitize the NLRP3 inflammasome-activating potential of macrophages but not neutrophils.

### Neutrophils Are Resistant to Inflammasome-Dependent Gasdermin D Cleavage and DAMP-Induced Mitochondrial Depolarization

**Figure 3:**
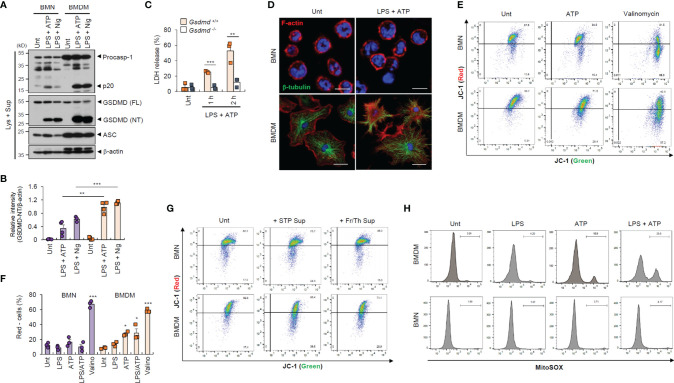
Neutrophils exhibit weaker gasdermin D cleavage and mitochondrial depolarization than macrophages in response to NLRP3 stimulation. **(A)** Immunoblots from mouse BMNs or BMDMs untreated (Unt) or primed with LPS (0.25 μg/mL, 2.5 h), followed by ATP (2.5 mM, 1h) or nigericin (Nig, 5 μM, 1 h) treatment. **(B)** Quantification of relative GSDMD (NT) band intensity versus β-actin band in **(A)**. (*n* = 4). **(C)** Quantification of LDH release into the culture supernatants of wild-type or *Gsdmd*-deficient mouse BMDMs primed with LPS followed by ATP (2.5 mM, 1 or 2 h) treatment (*n* = 3). **(D)** Representative immunofluorescence images of mouse BMNs or BMDMs treated with LPS followed by ATP (2 mM, 1 h) treatment after staining with anti-F-actin (red) and anti-α-tubulin (green) antibodies. Nuclei were stained with DAPI (blue). Scale bars, 10 μm (BMNs) or 20 μm (BMDMs). **(E)** Flow cytometric analysis of mouse BMNs or BMDMs treated with ATP (2.5 mM, 30 min) or valinomycin (5 μM, 30 min) treatment after JC-1 staining. **(F)** Quantification of JC-1 (red)-negative mouse BMDM or BMN populations treated with LPS alone, ATP alone, LPS followed by ATP or valinomycin (*n* = 3). **(G)** Flow cytometric analysis of mouse BMDMs or BMNs treated with cell-free supernatants of untreated cells or those injured by staurosporine treatment or repeated freeze/thaw cycles for 1.5 h after JC-1 staining. **(H)** Flow cytometric analysis of mouse BMDMs or BMNs treated with LPS alone (0.25 μg/mL, 2.5 h), ATP alone (2.5 mM, 0.5 h) or LPS followed by ATP treatment after MitoSOX staining. GSDMD; gasdermin D, FL; full length, NT; N-terminal. *P < 0.05, **P < 0.01, ***P < 0.001.

To provide a molecular insight into the distinct resistance of neutrophil to pyroptosis and NLRP3 desensitization, we first measured inflammasome component expression. We found that neutrophils displayed lower active caspase-1 (p20) levels than macrophages (**Figure 3A**), but similar levels of active IL-1β secretion (**Supplementary Figure S5A**). Likewise, GSDMD cleavage was much weaker in neutrophils than in macrophages (**Figures 3A, B** and **Supplementary Figure S5B**). These findings suggest that impaired GSDMD cleavage led to reduced GSDMD pore formation in the neutrophil plasma membrane. Indeed, GSDMD deficiency completely abrogated LPS/ATP-induced cell death in macrophages (**Figure 3C**). Moreover, LPS/ATP stimulation substantially disrupted plasma membrane integrity in macrophages but not neutrophils (**Figure 3D**). Consistently, LPS/ATP stimulation significantly increased membrane permeability and phosphatidylserine exposure in macrophages but much less in neutrophils (**Figures 1F, H**). These results indicate that lower GSDMD pore formation may partly explain the pyroptosis resistance of neutrophils.

Mitochondria are important signaling organelles in regulating NLRP3 inflammasome activation and many types of cell death (24, 25). To explore whether mitochondrial alterations are involved in the distinct pyroptosis resistance of neutrophils, we examined mitochondrial phenotype under inflammasome-activating conditions. Unexpectedly, ATP treatment and LPS/ATP stimulation led to profound mitochondrial depolarization in macrophages but not neutrophils, as indicated by an increase in the JC-1 (red)-negative population (**Figures 3E, F**). The selective caspase-1 inhibitor, ac-YVAD-cmk, did not protect macrophages against LPS/ATP-promoted mitochondria depolarization (**Supplementary Figure S6**), indicating that ATP treatment, but not active caspase-1, primarily caused the loss of mitochondrial membrane potential in macrophages. In neutrophils, mitochondrial depolarization was detected by valinomycin, widely used to cause the loss of mitochondrial membrane potential, but not by LPS/ATP stimulation (**Figures 3E, F**). Like ATP treatment, the DAMP-rich medium markedly impaired the mitochondrial membrane potential of macrophages but not neutrophils (**Figure 3G**). Moreover, ATP treatment induced a profound mitochondrial reactive oxygen species (ROS) production only in macrophages (**Figure 3H**), suggesting that DAMP or ATP may damage macrophage mitochondrial integrity but not that of neutrophils.

### Distinct Resistance of Neutrophils to Pyroptosis and NLRP3 Desensitization Is Associated With the Absence of SARM1-Induced Mitochondrial Depolarization

**Figure 4:**
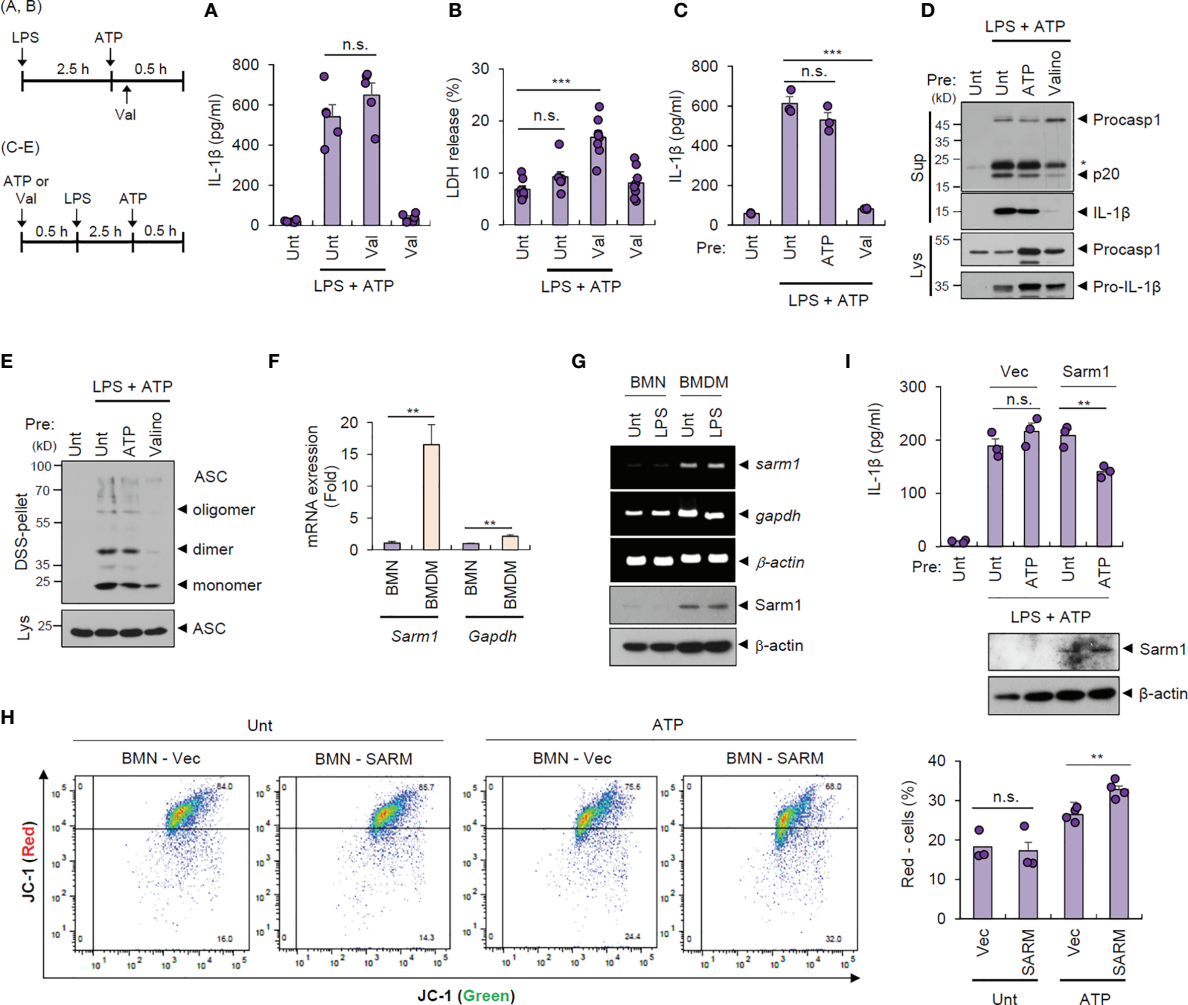
Cell type-specific changes in mitochondrial integrity determine pyroptosis resistance and NLRP3 desensitization. **(A, B)** Quantification of IL-1β **(A)** or LDH **(B)** release into the culture supernatant from mouse BMNs primed with LPS (0.25 μg/ml, 2.5 h) followed by ATP (2.5 mM, 1 h) treatment in the presence of valinomycin (10 μM, last 50 min) (*n* = 5 or 8). **(C, D)** Quantification of IL-1β in the culture supernatant **(C)** or immunoblots from mouse BMNs pretreated with ATP (2.5 mM) or valinomycin (10 μM) for 30 min, washed and primed with LPS (0.25 μg/mL, 2.5 h) followed by ATP (2.5 mM, 30 min) treatment (*n* = 3). The asterisk indicates a nonspecific band **(D)**. **(E)** Immunoblots of DSS-crosslinked pellets (DSS-pel) or cell lysates (Lys) from BMNs treated as in **(C, D)**. **(F)** Quantification of *sarm1* or *gapdh* mRNA levels in mouse BMNs or BMDMs (*n* = 5). **(G)** RT-PCR analysis and immunoblots of *sarm1* or *gapdh* in mouse BMNs or BMDMs untreated or treated with LPS (0.5 μg/mL, 3 h). **(H)** Mouse BMNs were transfected with vector or Sarm1-expressing construct using DOTAP liposomal reagent and then treated with ATP (2.5 mM, 1 h). Flow cytometric analysis of these cells after JC-1 staining (left panel). Quantification of JC-1 (red)-negative mouse BMN populations (right panel, *n* = 3 or 4). **(I)** Quantification of IL-1β (upper panel) or immunoblots (lower panel) of mouse BMNs transfected with empty vector or Sarm1-expressing construct, and treated with ATP (2 mM, 20 min), and then treated with LPS followed by ATP treatment. (*n* = 3). Culture supernatants (Sup) or cell lysates (Lys) were immunoblotted with the indicated antibodies. **P < 0.01, ***P < 0.001, n.s., not significant.

To examine whether intact mitochondrial membrane potential can explain the unique resistance of neutrophils to pyroptosis and NLRP3 desensitization, we treated cells with valinomycin to induce compulsory mitochondrial depolarization. Interestingly, valinomycin treatment (10 min after ATP treatment) did not affect LPS/ATP-promoted IL-1β secretion by neutrophils (**Figure 4A**) but caused a significant pyroptosis in neutrophils (**Figure 4B**), indicating that mitochondrial membrane potential plays a key role in determining pyroptosis resistance. Moreover, pretreatment with valinomycin significantly abolished NLRP3 inflammasome activation in neutrophils, while ATP pretreatment failed to attenuate LPS/ATP-triggered IL-1β secretion (**Figure 4C**). Consistently, rotenone, another chemical to cause mitochondrial depolarization, pretreatment led to impaired IL-1β production by neutrophils (**Supplementary Figure S7**). These observations suggest that intact mitochondrial membrane potential at the moment of second signal, such as ATP or nigericin, is prerequisite for normal NLRP3 inflammasome-activating potential. Accordingly, valinomycin pretreatment clearly diminished LPS/ATP-triggered caspase-1 activation (**Figure 4D**) and ASC oligomerization in neutrophils (**Figure 4E**).

We were then keen to unveil the mechanism underlying the different status of the mitochondrial membrane potential of both cells exposed to the DAMP- or ATP-rich milieu. A recent study demonstrates that Sterile α- and heat armadillo motif-containing protein 1 (SARM1) clustering contributes to the induction of mitochondrial depolarization upon treatment with NLRP3 stimulants (26). Intriguingly, neutrophils displayed negligible Sarm1 expression compared to macrophages (**Figures 4F, G**). We thus speculate that the absence of Sarm1 expression is implicated in the neutrophil resistance to mitochondrial depolarization by ATP stimulation. Indeed, liposome-mediated Sarm1 transfection caused a considerable increase in the ATP-driven mitochondrial depolarization in neutrophils (**Figure 4H**). Sarm1 transfection did not impair cell viability of neutrophils (**Supplementary Figure S8**), but significantly diminished IL-1β secretion from neutrophils in response to the following LPS/ATP stimulation (**Figure 4I**). These results indicate that sarm1-deficiency is potentially implicated in the neutrophil resistance to NLRP3 desensitization.

### Neutrophils Drive Sustained IL-1β-Specialized Inflammatory Responses

As shown above, neutrophils exhibited much lower caspase-1 cleavage than macrophages, but equivalent IL-1β secretion. To determine whether a protease other than caspase-1 was implicated in IL-1β processing and secretion in neutrophils, we measured IL-1β production in the presence of the caspase-1-selective inhibitor, YVAD. In neutrophils, YVAD markedly blocked active IL-1β production in response to NLRP3 stimulation (**Supplementary Figures S9A, B**). Moreover, NLRP3 was essential for ATP- or nigericin-triggered IL-1β secretion by neutrophils (**Supplementary Figure S9C**), indicating that IL-1β secretion by neutrophil is NLRP3/caspase-1-dependent similar to macrophages.

**Figure 5:**
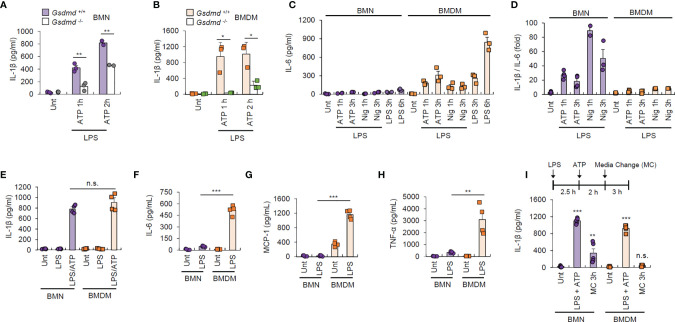
Neutrophils exhibit unique IL-1β-oriented cytokine production. **(A, B)** Quantification of IL-1β in the culture supernatant of *Gsdmd* ^+/+^ or *Gsdmd* ^-/-^ mouse BMNs **(A)** or BMDMs **(B)** treated with LPS (0.25 μg/mL, 2.5 h) followed by ATP (2.5 mM, 1 or 2 h) treatment (*n* = 3). **(C, D)** Quantification of IL-6 **(C)** or the relative IL-1β/IL-6 ratio **(D)** in the culture supernatant of mouse BMNs or BMDMs primed with LPS (0.25 μg/mL, 2.5 h), washed and treated with ATP (2.5 mM) or nigericin (5 μM) for 1 or 3 h, or treated with LPS alone (*n* = 3). **(E)** Quantification of IL-1β in the culture supernatant of mouse BMNs or BMDMs primed with LPS (0.25 μg/mL, 2.5 h) followed by ATP (2.5 mM, 1 h) treatment (*n* = 4). **(F–H)** Quantification of IL-6 **(F)**, MCP-1 **(G)**, and TNF-α **(H)** in the culture supernatant of mouse BMNs or BMDMs treated with LPS (0.25 μg/mL, 3 h) (*n* = 4). **(I)** Quantification of IL-1β in the culture supernatant of mouse BMNs or BMDMs treated with LPS (0.25 μg/mL, 2.5 h) followed by ATP (2.5 mM, 2 h) treatment, washed and incubated for 3 h in the presence of additional LPS and ATP (*n* = 5). Culture supernatants (Sup) or cell lysates (Lys) were immunoblotted with the indicated antibodies. *P < 0.05, **P < 0.01, ***P < 0.001, n.s. not significant.

GSDMD pore is the major conduit for IL-1β secretion in macrophages (27). As neutrophils displayed much weaker GSDMD pore formation than macrophages (**Figure 3**), we determined IL-1β secretion levels in wild-type and *Gsdmd*-deficient cells. GSDMD presence was essential for early IL-1β secretion by neutrophils, but partial GSDMD-independent IL-1β secretion was observed in neutrophils after 2 h of ATP stimulation (**Figures 5A, B**). Besides, neutrophils displayed more rapid IL-1β secretion than macrophages (**Supplementary Figure S9D**), suggesting that neutrophil may have a somewhat different IL-1β secretion mechanism to macrophages.

Intriguingly, we noticed that IL-6 production in response to NLRP3-activating or LPS stimulations was very low in neutrophils but robust in macrophages (**Figure 5C**). Indeed, neutrophils elicited more IL-1β-oriented production than macrophages under NLRP3 inflammasome-activating conditions (**Figure 5D**). Then, we measured the secretion of several cytokines from both cell types in response to LPS stimulation using cytokine-bead array experiments. LPS alone caused robust IL-6, MCP-1 and TNF-α production in macrophages, but none or much less from neutrophils, whereas IL-1β secretion was similar in both cell types following LPS/ATP stimulation (**Figures 5E–H**). Furthermore, neutrophils facilitated persistent IL-1β secretion after media change following 2 h of ATP stimulation, unlike macrophages (**Figure 5I**). Together, these data demonstrate that neutrophils can drive IL-1β–oriented inflammatory responses more persistently than macrophages.

### Inflammasome-Active Neutrophils Maintain Antimicrobial Functions but Do Not Trigger Efferocytosis-Mediated M2-Like Macrophage Polarization

**Figure 6:**
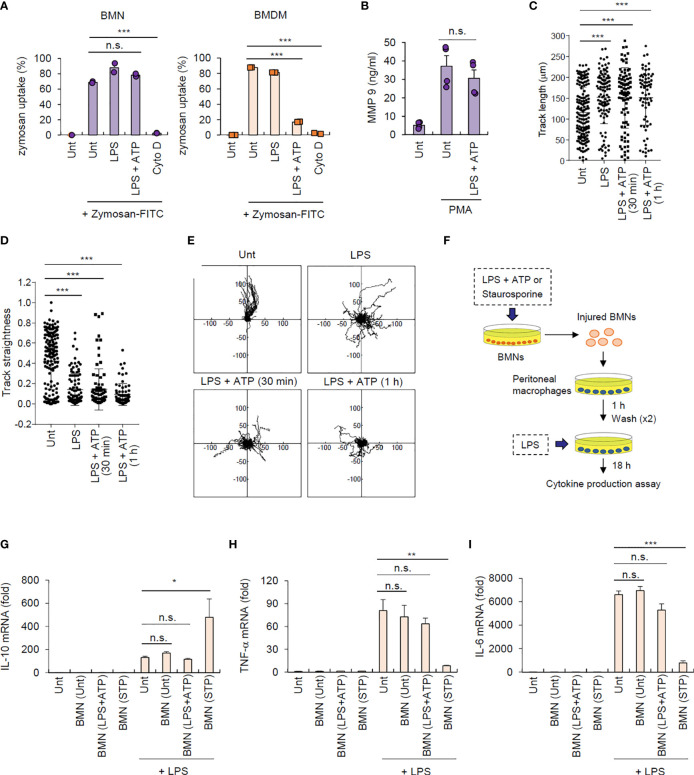
Inflammasome-active neutrophils maintain phagocytosis, degranulation, and migration activities, but do not trigger efferocytic anti-inflammatory macrophage polarization. **(A)** Mouse BMNs or BMDMs were untreated (Unt) or treated with LPS (0.25 μg/mL, 2.5 h) alone, LPS followed by ATP (2.5 mM, 1 h), or cytochalasin D (Cyto D, 10 μM, 30 min) as determined using flow cytometric analysis after incubating with zymosan-FITC (5 particles/cell, 30 min) (*n* = 3). **(B)** Quantification of MMP-9 in the culture supernatants of mouse BMNs treated with LPS (0.25 μg/mL, 2.5 h) followed by ATP (2.5 mM, 1 h), washed and treated with PMA (1 μM, 2 h) (*n* = 3). **(C, D)** Quantification of track length **(C)** and track straightness **(D)** of BMNs treated with LPS (0.25 μg/mL, 2.5 h) alone or followed by ATP (2.5 mM), as determined using *in vitro* migration assays. **(E)** Representative migration tracks of BMNs treated as in **(C, D)**. **(F)** Experimental scheme for determining the potential effect of inflammasome-active neutrophils on efferocytosis. **(G–I)** Mouse BMNs were untreated or treated with LPS (0.25 μg/mL, 2.5 h) followed by ATP (2.5 mM, 2 h) or staurosporine (2 μg/mL, 5 h). Mouse peritoneal macrophages were treated with the injured BMNs (1:2.5 ratio, macrophages:neutrophils) for 1 h, washed and treated with LPS (0.1 μg/mL) for 18 **(H)** Quantification of *IL-10* **(G)**, *TNF-α* **(H)**, or *Il-6* **(I)** mRNA levels in mouse peritoneal macrophages treated as above (*n* = 3). *P < 0.05, **P < 0.01, ***P < 0.001, n.s. not significant.

Next, we determined the traditional function of neutrophils after inflammasome activation. We examined the phagocytic activity of inflammasome-active neutrophils and macrophages using a zymosan phagocytosis assay. Cytochalasin D, an actin polymerization inhibitor, was used as a control for the inhibition of phagocytosis. NLRP3 inflammasome-activating LPS/ATP stimulation did not affect zymosan uptake in neutrophils but markedly impaired this process in macrophages (**Figure 6A** and **Supplementary Figure S10**), indicating that inflammasome activation may impair the phagocytic activity of macrophages but not neutrophils. We also examined whether inflammasome activation can regulate neutrophil degranulation activity. Consistent with its effect on phagocytosis, NLRP3 inflammasome activation did not significantly affect neutrophil degranulation by measuring the extracellular release of matrix metallopeptidase 9 (MMP-9) triggered by phorbol-12-myristate-13-acetate (PMA) (**Figure 6B**).

Additionally, we measured the migration ability of neutrophils after inflammasome activation using an *in vitro* migration assay. Interestingly, LPS/ATP stimulation did not alter neutrophil migration speed (**Supplementary Figure S11**) but significantly increased their total migration track length (**Figure 6C**). Of note, LPS/ATP treatment reduced the straightness of neutrophil migration (**Figure 6D**) and enhanced random migration patterns (**Figure 6E** and **Supplementary Movie 1**). Together, these findings indicate that inflammasome activation does not impair but slightly increase neutrophil migration ability.

As shown in **Figure 1H**, NLRP3 inflammasome activation caused much less phosphatidylserine exposure, a typical eat-me signal, in neutrophils than in macrophages. Dying neutrophils are engulfed by macrophages in a process known as efferocytosis, leading to M2-like macrophage polarization and inflammation resolution (4, 28). We thus examined whether inflammasome-active neutrophils could promote efferocytosis-mediated signaling of target macrophages (**Figure 6F**). Consistent with previous reports (28), staurosporine-treated neutrophils significantly increased IL-10 production by peritoneal macrophages stimulated with LPS (**Figure 6G**); however, LPS/ATP-stimulated neutrophils did not alter IL-10 production in macrophages (**Figure 6G**), indicating that inflammasome-active neutrophils do not induce efferocytosis-mediated M2 macrophage polarization. Supporting this finding, LPS/ATP-treated neutrophils failed to affect the production of proinflammatory cytokines, including TNF-α (**Figure 6H**) and IL-6 (**Figure 6I**), by peritoneal macrophages upon LPS stimulation, whereas staurosporine-stimulated neutrophils significantly abolished proinflammatory cytokine production in these macrophages. Together, these findings suggest that inflammasome-activated neutrophils do not immunologically silence target macrophages but instead mediates prolonged inflammation in the inflamed region.

### Neutrophils Resist Pyroptosis in LPS-Challenged *In Vivo* Mouse Model

**Figure 7:**
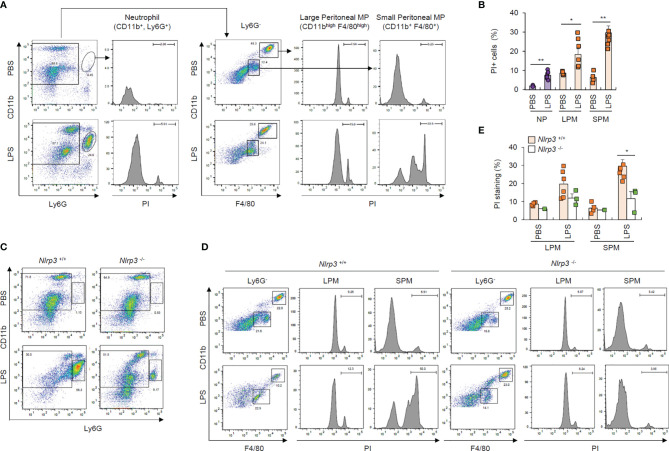
Neutrophils resist pyroptosis in an *in vivo* mouse model. **(A)** Flow cytometric analysis of peritoneal lavage from mice administered PBS or LPS (1 mg/kg, 24 h) after staining with anti-CD11b, Ly6G, and F4/80 antibodies and propidium iodide (PI). **(B)** Quantification of PI-positive cells of neutrophils (NP), large or small peritoneal macrophages (LPM or SPM) as indicated in **(A)** (*n* = 2~7). **(C, D)** Flow cytometric analysis of mouse peritoneal lavage from wild-type or *Nlrp3*-deficient mice administered PBS or LPS (1 mg/kg, 24 h) after staining with anti-CD11b, Ly6G, and F4/80 antibodies and PI. **(E)** Quantification of PI-positive cells of LPM or SPM as treated in **(D)** (*n* = 2~7). *P < 0.05, **P < 0.01.

To confirm pyroptosis resistance in neutrophils under physiological conditions, we intraperitoneally injected mice with PBS or LPS and analyzed cells in the peritoneal lavage after 24 h of LPS challenge. We found that considerably more Ly6G^+^ neutrophils were isolated from the peritoneal lavage fluid of LPS-treated mice than PBS-treated mice (**Figure 7A**). Although PI-stained dead neutrophils population was less than 10%, even in the LPS-treated mice (**Figure 7B**), LPS administration dramatically increased the population of PI-positive dead small and large peritoneal macrophages (**Figures 7A, B**). Thus, these *in vivo* data indicate that LPS challenge causes significant cell death in macrophages from peritoneal lavage fluid, but much less cell death in neutrophils.

To examine whether the above macrophage cell death was inflammasome-dependent, we performed the experiments using wild-type and *Nlrp3*-deficient mice. Notably, the LPS-driven increase in the Ly6G^+^ neutrophils in the peritoneal lavage of wild-type mice was significantly diminished in the peritoneal lavage of NLRP3-knockout mice (**Figure 7C**), while LPS administration profoundly increased cell death in small macrophages from the peritoneal lavage of wild-type mice compared to *Nlrp3*-deficient mice (**Figures 7D, E**). These *in vivo* results indicate that NLRP3 inflammasome-activating conditions lead to the apparent NLRP3-dependent cell death of macrophages under physiological conditions.

## Discussion

Chronic and sustained inflammation or inflammasome activation can be detrimental to host tissues and exacerbates the pathogenesis of diverse inflammatory or metabolic diseases (29). However, inflammasome assembly promptly induces the caspase-1 or caspase-11-dependent GSDMD cleavage and subsequent GSDMD pore formation in the plasma membrane leading to pyroptosis (30). The pyroptosis of inflammasome-activated cells can, therefore, terminate the inflammasome response and IL-1β production in the inflammatory region to prevent sustained inflammasome activation. Consistently, previous studies have demonstrated that pyroptotic macrophages lose their functional integrity upon inflammasome activation (31). Pyroptosis occurs in various inflammasome-active cell types, including macrophages, monocytes, dendritic cells and endothelial cells (32–34); therefore, it remains unclear which cell types can overcome pyroptosis and facilitate sustained inflammasome responses in chronic inflammasome-related diseases.

Consistent with recent reports (9, 14, 15, 35), we clarified pyroptosis resistance of neutrophils using diverse *in vitro* experiments and *in vivo* conditions. Molecular mechanisms underlying the pyroptosis resistance of neutrophils remain elusive. It was previously suggested that smaller ASC speck in neutrophils might implicate pyroptosis resistance (36). In the present study, our data revealed that neutrophils exhibited weaker caspase-1 processing and GSDMD cleavage than macrophages, which led to the reduced number of GSDMD pores in the plasma membrane of neutrophils, thereby affecting their resistance to pyroptosis. Of interest, a recent study demonstrated that cleaved GSDMD does not localize to the plasma membrane, but migrates to azurophilic granules in neutrophils (37), supporting our hypothesis that neutrophils exhibit impaired GSDMD pore formation in the plasma membrane. On the other hand, our data also indicate that mitochondrial membrane potential is critical for determining pyroptosis. Carty et al. recently reported that SARM1-dependent mitochondrial depolarization regulates pyroptosis in macrophages (26). Interestingly, we found that SARM1 expression was not observed in neutrophils and the preservation of mitochondrial membrane potential under NLRP3-activating stimulation contributes to the pyroptosis resistance of neutrophils.

GSDMD pore in the plasma membrane has been shown to act as a major conduit for IL-1β secretion in macrophages (27). Despite reduced GSDMD pore formation, neutrophils displayed similar levels of IL-1β secretion to macrophages. At present, the mechanism underlying this phenomenon is not yet completely understood. Our data demonstrated that neutrophil IL-1β secretion is still caspase-1 activity- and GSDMD presence-dependent. A recent study proposed that N-GSDMD localizes to azurophilic granules and autophagosomes in neutrophils rather than the plasma membrane (37), with this unique pattern of GSDMD trafficking driving IL-1β secretion in a GSDMD/ATG7-dependent manner. In accordance with this finding, our current study supports the possibility that neutrophil IL-1β secretion depends on GSDMD but not GSDMD pores in the plasma membrane. Of interest, a slower GSDMD-independent IL-1β secretion was recently proposed in the non-pyroptotic cells such as neutrophils (38). Consistently, we also found that some GSDMD-independent IL-1β release was observed after 2 h of ATP stimulation.

We also demonstrated that neutrophils induced more rapid IL-1β secretion than macrophages in response to NLRP3 activation. Since rapid IL-1β secretion was found to involve microvesicle shedding in THP-1 monocytes (39), it is possible that neutrophils may also use microvesicle shedding rather than GSDMD pore for very early IL-1β secretion following NLRP3 stimulation. Therefore, the detailed molecular mechanism *via* which neutrophils secret IL-1β after NLRP3 inflammasome activation remains to be further elucidated. Interestingly, our study revealed that neutrophils predominantly secrete IL-1β rather than other proinflammatory cytokines upon NLRP3 stimulation. Similarly, previous studies demonstrated that neutrophils produce much lower levels of proinflammatory cytokines in response to diverse types of stimulation (40, 41). Moreover, Bakele et al. showed that NLRP3 activation caused a robust IL-1β, but not IL-18 secretion by neutrophils, while macrophages produced both cytokines (42). Based on these observations, we propose that neutrophils can act as specialized IL-1β-producing cells under NLRP3 agonist-rich circumstances.

During the initial phase of inflammation, some resident cells are injured or die, leading to the release of cellular contents that produce a danger signal-rich milieu (21). To maintain prolonged inflammasome activation in such inflamed tissues, recruited fresh myeloid cells must promote a robust inflammasome response. Interestingly, we noticed that danger signal-rich medium or ATP clearly abrogated the NLRP3 inflammasome-activating capacity of macrophages. This observation is consistent with a recent study demonstrating that ATP-P2X7 signaling compromises NLRP3 inflammasome activation in macrophages (43). Unexpectedly, our study revealed that danger signal or ATP pretreatment failed to mitigate NLRP3-activating potential of neutrophils, strongly suggesting that recruited or infiltrated neutrophils can activate NLRP3 inflammasome signaling in danger signal-rich inflamed tissues and that neutrophils are major inflammasome-activating cells, particularly under ATP-rich conditions (**Supplementary Figure S12**).

Although the detailed molecular mechanism for the desensitization of NLRP3 inflammasome by danger signals remains unclear, we found that mitochondrial membrane potential status is closely associated with this phenomenon. Indeed, pretreatment with danger signal-rich medium or ATP caused mitochondrial depolarization in macrophages but not in neutrophils, while valinomycin-induced mitochondrial depolarization significantly abolished the NLRP3 inflammasome-activating potential of neutrophils. SARM is a potent candidate marker for macrophage-specific mitochondrial depolarization to determine cellular fate after inflammasome activation (26). Our data revealed that no robust SARM expression was detected in neutrophils. We also found that intact mitochondrial membrane potential is vital for the assembly and activation of NLRP3 inflammasome during treatment with NLRP3-activating second signals such as ATP or nigericin. These results suggest that intact mitochondria may act as a molecular platform for NLRP3 inflammasome assembly.

In addition to this sustained inflammasome-activating potential of neutrophils, our data further indicate that inflammasome-activated neutrophils do not induce efferocytosis-mediated M2-like macrophage polarization. Previous reports have shown that inflammasome-activated macrophages can trigger efferocytosis and downstream signaling of engulfed macrophages *via* exposing eat-me and find-me signals (44, 45). In our study, phosphatidylserine exposure, a typical eat-me signal, was unequivocally observed in inflammasome-activated macrophages, but not neutrophils. These findings indicate that inflammasome-active macrophages can contribute to efferocytosis-mediated inflammation resolution, while inflammasome-active neutrophils do not support efferocytosis but rather facilitate prolonged inflammasome responses (**Supplementary Figure S12**).

Taken together, our data provide new insights into the physiological role of neutrophil inflammasome signaling. In particular, distinct resistance of neutrophils to pyroptosis and DAMP-induced NLRP3 desensitization can prolong robust NLRP3 inflammasome response in danger signal-rich milieu during the propagation stage of inflammation. Thus, our results highlight the importance of neutrophils in the persistent inflammasome activation in response to chronic insult of endogenous metabolites, but not to acute microbial infection. Furthermore, our data suggest that this prolonged inflammasome-activating potential of neutrophils may contribute to the pathogenesis of chronic neutrophil-mediated inflammatory diseases.

## Data Availability Statement

The original contributions presented in the study are included in the article/**Supplementary Material**. Further inquiries can be directed to the corresponding author.

## Ethics Statement

The animal study was reviewed and approved by Institutional Ethical Committee, Yonsei University College of Medicine.

## Author Contributions

SS, S-HY, BC, IH, and D-WS performed experiments and analyzed the data. YC and Y-MH performed and analyzed the experiments regarding neutrophil migration. J-WY supervised the entire project. SS and J-WY wrote the manuscript. All authors contributed to the article and approved the submitted version.

## Funding

This work was supported by the National Research Foundation of Korea Grant funded by the Korean Government (2017R1A2B2007467, 2020R1A2B5B02001823, 2020R1A4A1019009) and the “Dongwha” Faculty Research Assistance Program of Yonsei University College of Medicine (6-2019-0122).

## Conflict of Interest

The authors declare that the research was conducted in the absence of any commercial or financial relationships that could be construed as a potential conflict of interest.

## Publisher’s Note

All claims expressed in this article are solely those of the authors and do not necessarily represent those of their affiliated organizations, or those of the publisher, the editors and the reviewers. Any product that may be evaluated in this article, or claim that may be made by its manufacturer, is not guaranteed or endorsed by the publisher.
