# Supplementary material for: Neutrophils Facilitate Prolonged Inflammasome Response in the DAMP-Rich Inflammatory Milieu
Source: Front Immunol. 2021 Sep 29;12:746032. doi: 10.3389/fimmu.2021.746032 (PMC8511454; doi:10.3389/fimmu.2021.746032)
Supplement: Supplementary file 1 [file DataSheet_1.pdf]

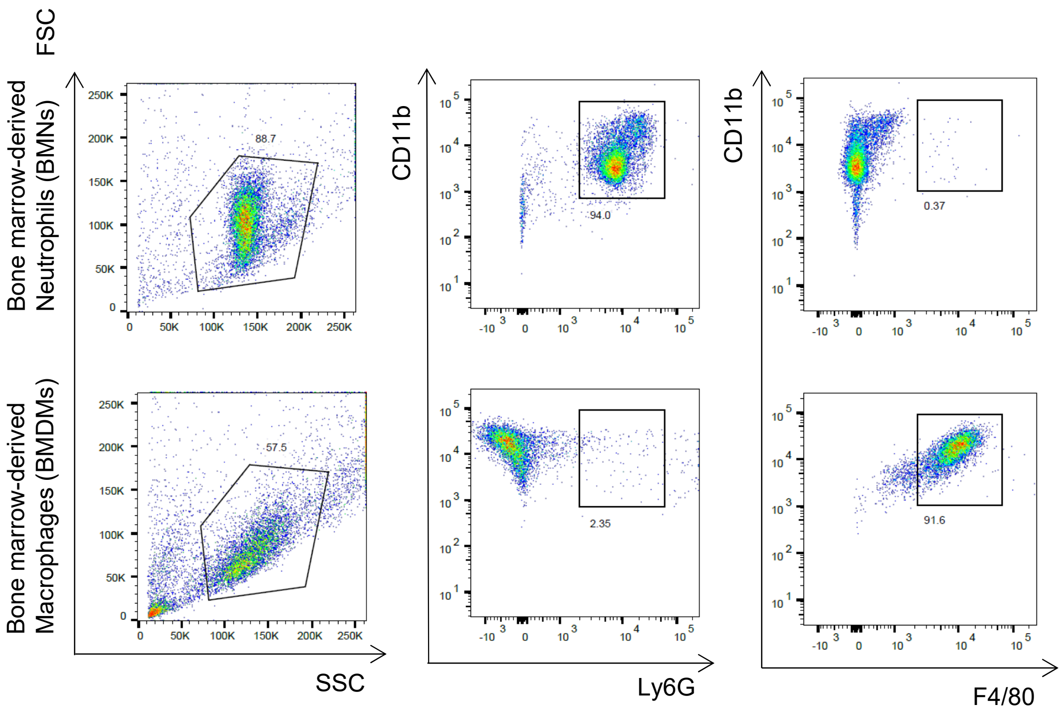

Supplementary Figure S1. Flow cytometric analysis of mouse bone marrow-derived neutrophils and macrophages. Mouse bone marrow-derived neutrophils or macrophages were stained with anti-CD11b, Ly6G and F4/80 antibodies and the fluorescence of the cells was then monitored and analyzed by flow cytometry.

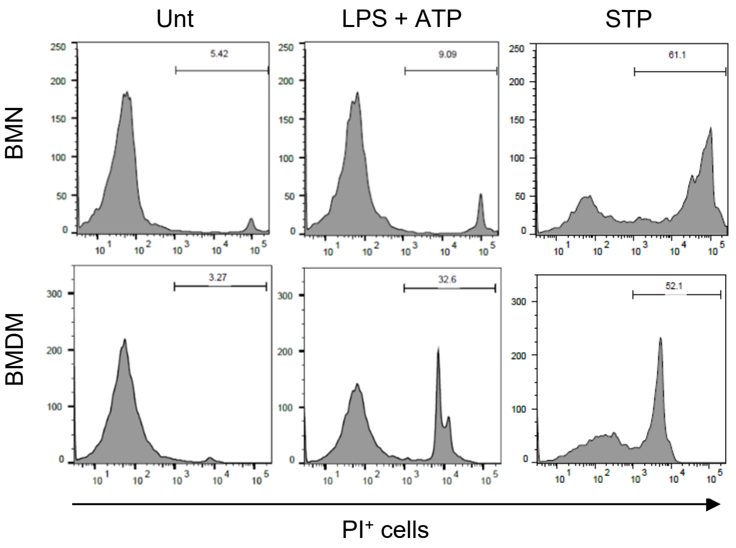

Supplementary Figure S2. Cell death analysis of neutrophils and macrophages after LPS/ATP or staurosporine stimulation. Representative flow cytometric diagram of mouse BMNs or BMDMs primed with LPS (0.25 μg/mL, 2.5 h) followed by ATP (2.5 mM, 1 h) treament or staurosporine (STP, 2 μg/mL, 6 h) treatment after propidium iodide (PI) staining.

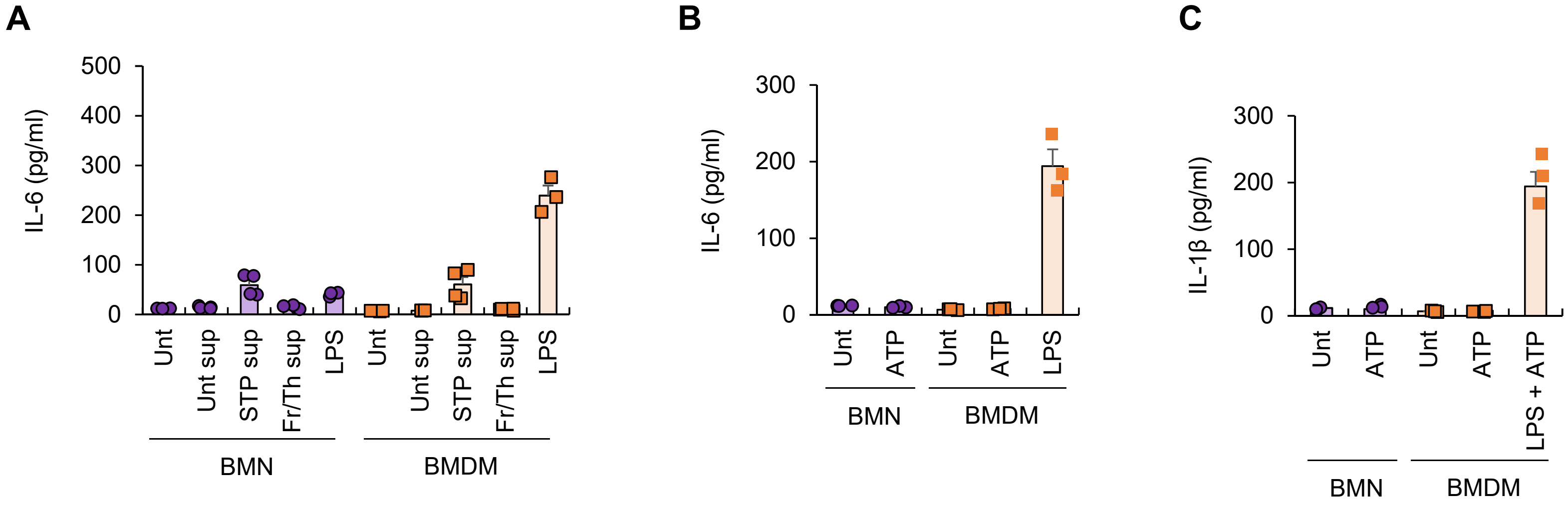

Supplementary Figure S3. Impaired IL-6 production from neutrophils. (A) Quantification of IL-6 in the culture supernatants of mouse bone marrow-derived neutrophils (BMNs) or bone marrow-derived macrophages (BMDMs) treated with cell-free supernatants from untreated or injured BMDMs by staurosporine (STP) or repeated freeze/thaw (Fr/Tha) cycles, or treated with LPS (0.25 μg/ml, 3 h). (*n* = 3) **(B)** Quantification of IL-6 in the culture supernatants of mouse BMNs or BMDMs treated with ATP (2.5 mM, 1 h) or LPS (0.25 μg/ml, 3 h). (*n* = 3) **(C)** Quantification of IL-1β in the culture supernatants of mouse BMNs or BMDMs treated with ATP (2.5 mM, 1 h) or LPS (0.25 μg/ml, 3 h), followed by ATP (2.5 mM, 30 min). (*n* = 3)

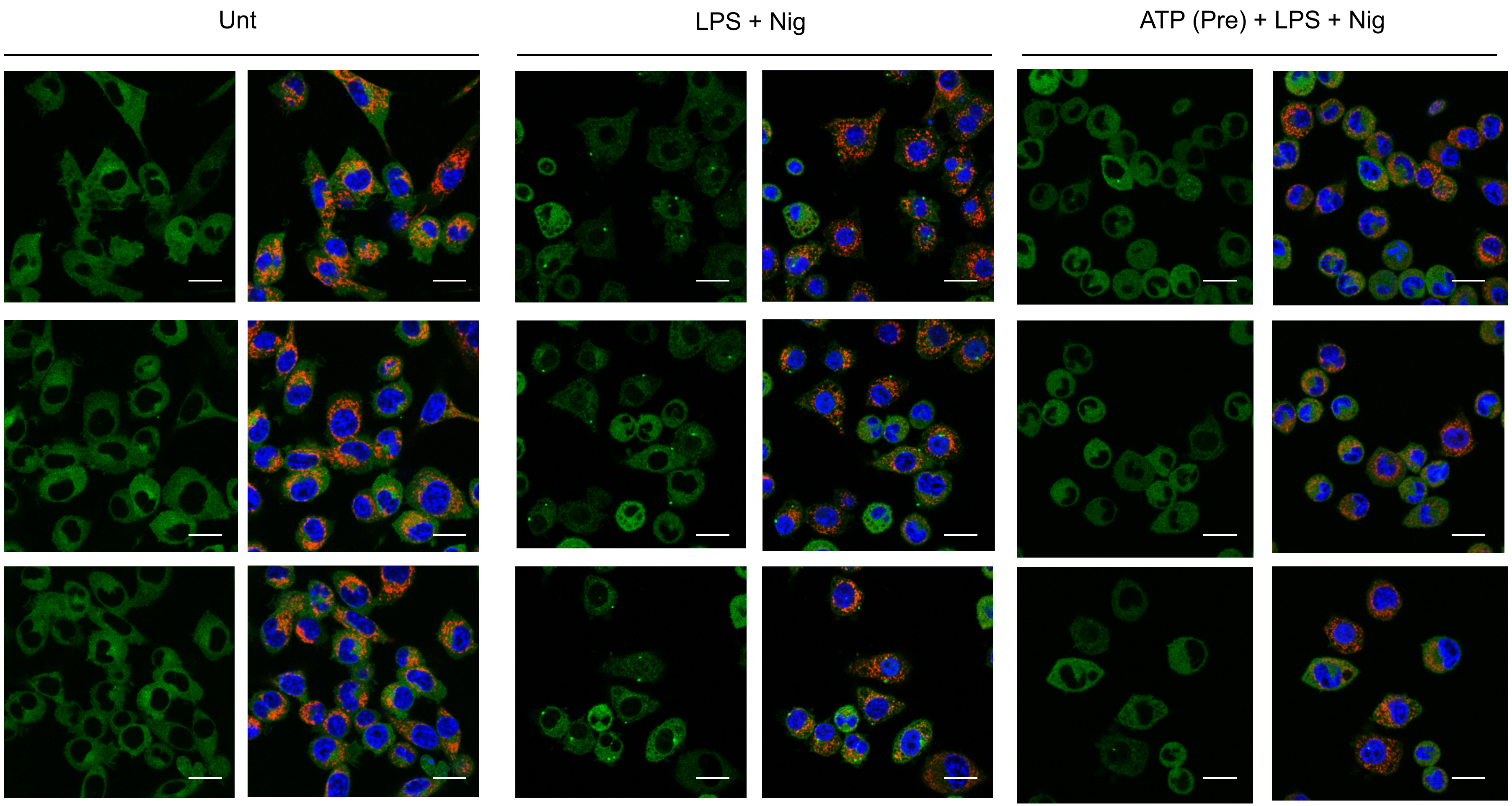

Supplementary Figure S4. Effect of ATP pretreatment on the NLRP3 oligomerization in mouse bone marrow-derived macrophages. Representative immunofluoerscent images of NLRP3-GFP-expressing immortalized BMDMs pretreated with ATP (2 mM, 30 min), washed and treated with LPS (0.25 μg/ml, 2.5 h), followed by nigericin (Nig, 5 μM, 30 min) treatment after staining with anti-Tom20 antibody (red). DAPI represents the nuclear signal (blue). Scale bars, 20 μm.

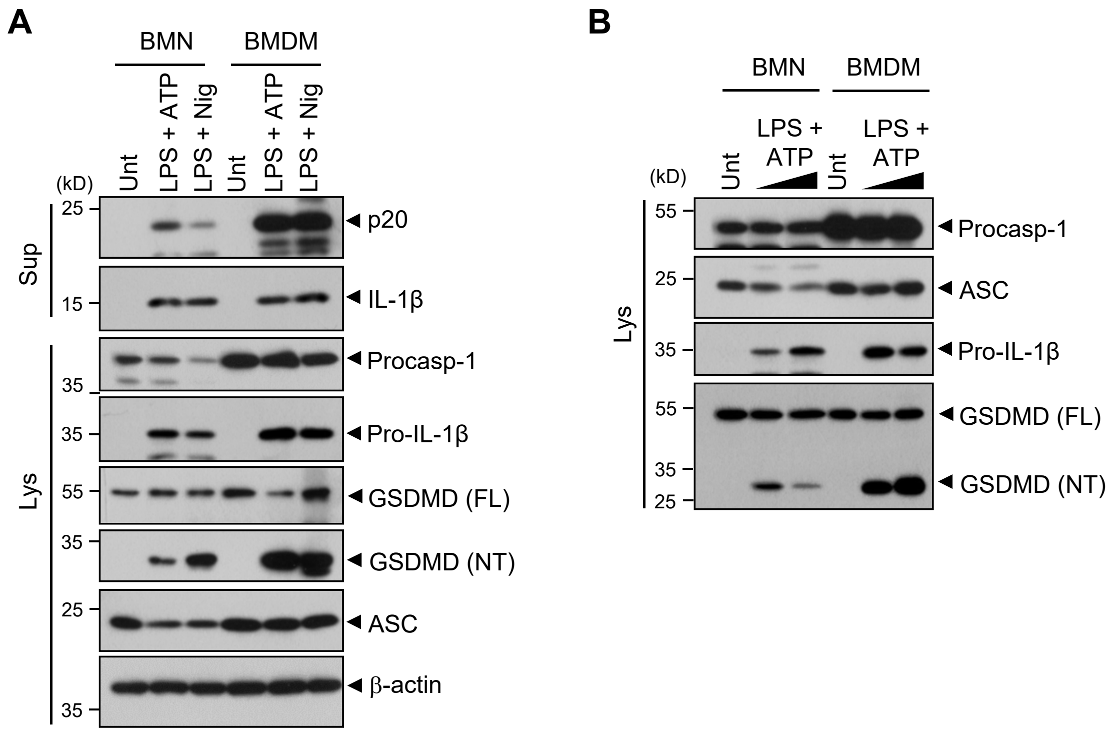

Supplementary Figure S5. Immunoblots from neutrophils or macrophages in response to NLRP3-activating stimulations. (**A**) Mouse neutrophils or macrophages were primed with LPS (0.25 μg/ml, 2.5 h) followed by ATP (2.5 mM, 1 h) or nigericin (5 μM, 1 h) treatment. (**B**) Mouse neutrophils or macrophages were primed with LPS (0.25 μg/ml, 2.5 h) followed by ATP (2.5 mM, 1 or 3 h) treatment. Culture supernatants (Sup) or cellular lysates (Lys) were immunoblotted with the indicated antibodies. GSDMD; gasdermin D, FL; full length, NT; N-terminal.

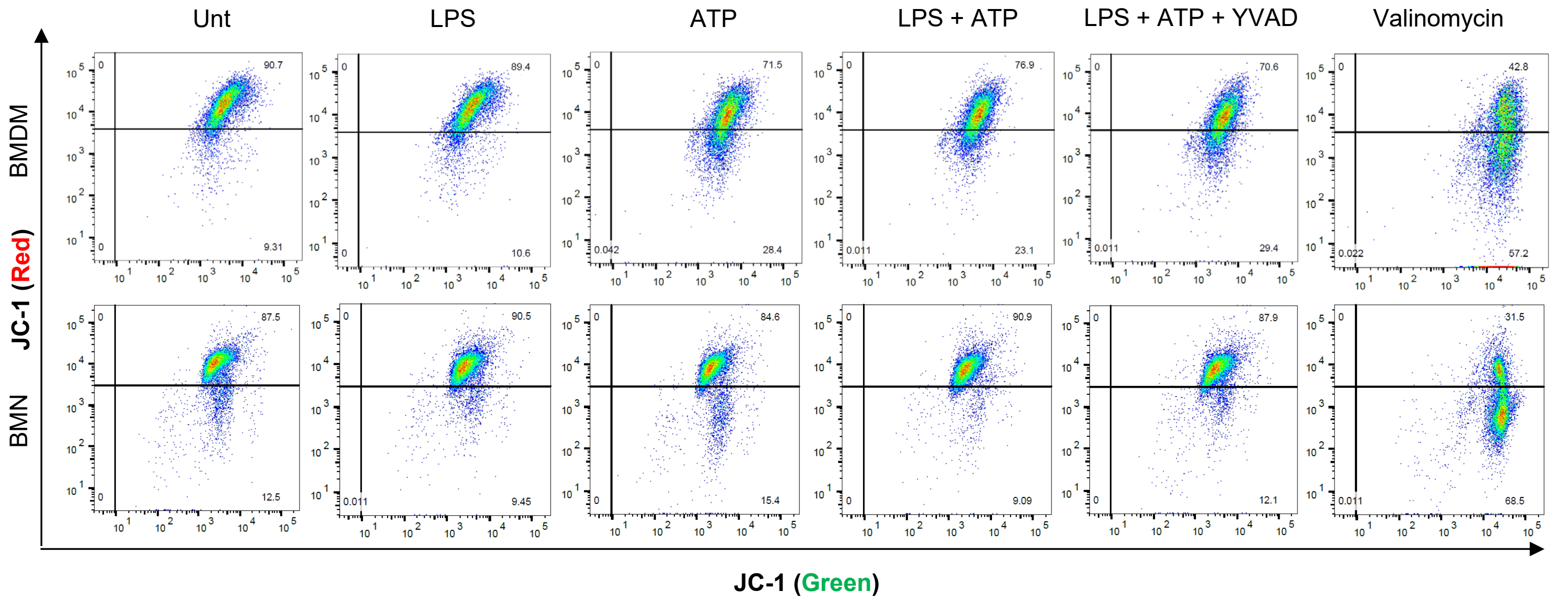

Supplementary Figure S6. Mitochondrial membrane potential of neutrophils or macrophages in response to NLRP3-activating stimulations. Flow cytometric analysis of mouse BMNs or BMDMs treated with LPS (0.25 µg/mL) alone, ATP (2.5 mM) alone, LPS followed by ATP treatment in the presence of YVAD (20 µM) or valinomycin treatment (5 µM) after JC-1 staining.

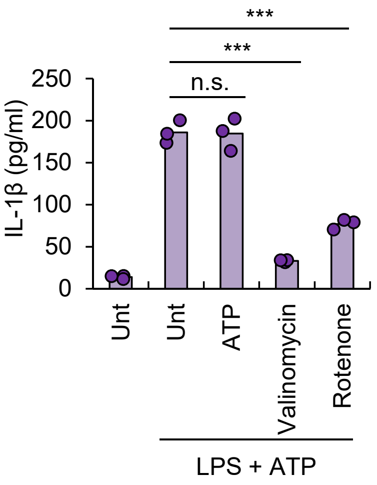

Supplementary Figure S7. Effect of mitochondrial depolarization on the NLRP3-activating potential in neutrophils. Quantification of IL-1β in the culture supernatant from mouse BMNs pretreated with ATP (2.5 mM), valinomycin (5 μM) or rotenone (5 μM) for 30 min, washed and primed with LPS (0.25 μg/mL, 2.5 h) followed by ATP (2.5 mM, 30 min) treatment (n = 3). ***P < 0.001, n.s. not significant.

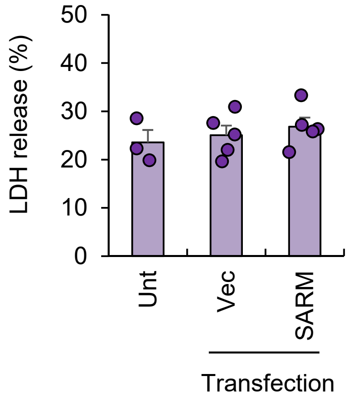

Supplementary Figure S8. Cell death analysis of neutrophils after transfection. Quantification of LDH release into cell culture supernatants of bone marrow-derived neutrophils transfected with empty vector (Vec) or Sarm1-expressing construct (SARM) for 22 h. (*n* = 3 ~ 5)

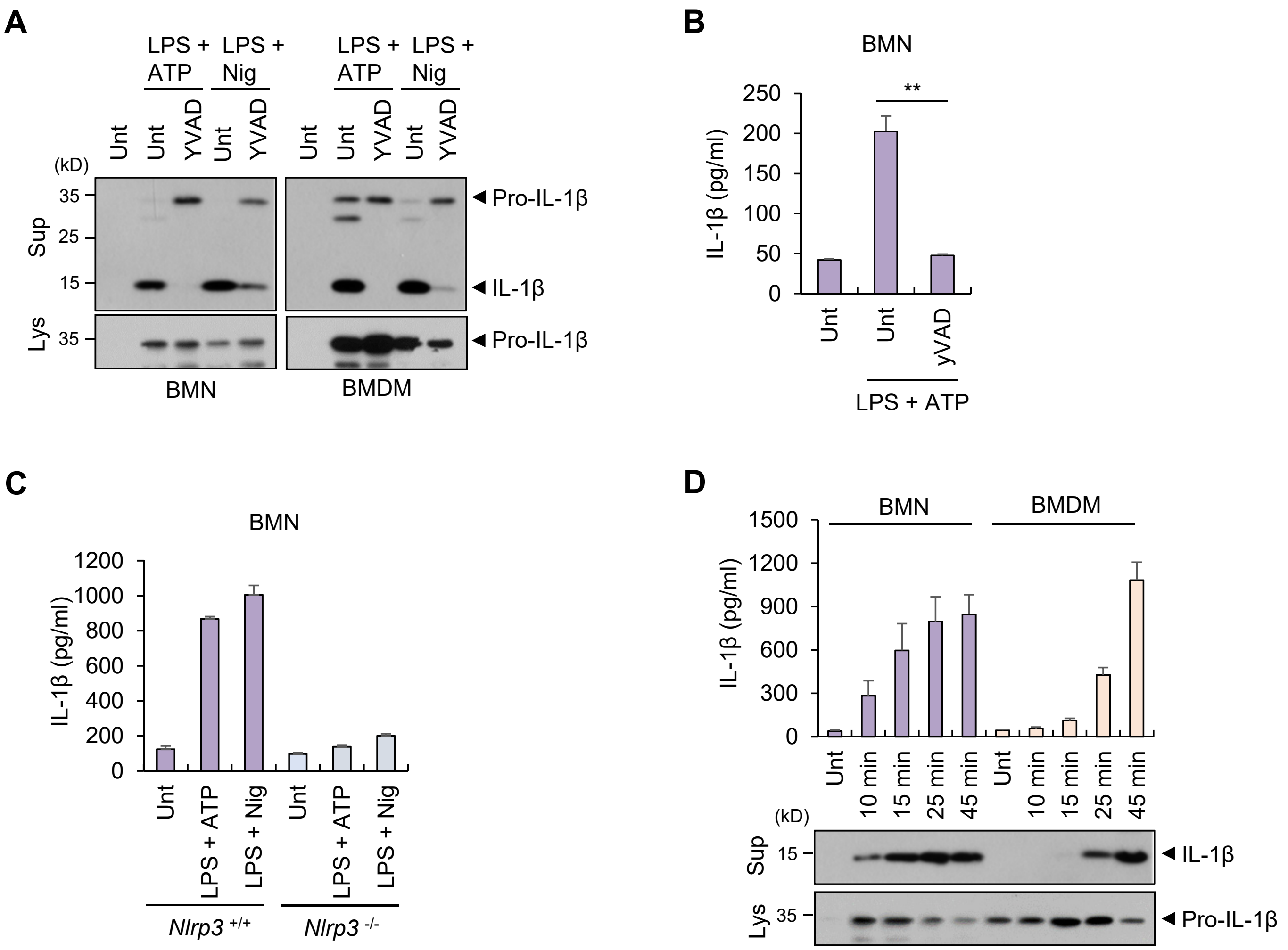

Supplementary Figure S9. Neutrophils exhibit caspase-1- and NLRP3-dependent IL-1β processing and secretion. (**A**) Immunoblots of mouse BMNs or BMDMs primed with LPS (0.25 μg/mL) followed by ATP (2.5 mM) or nigericin (5 μM) treatment in the presence of ac-YVAD-cmk (20 μM). (**B**) Quantification of IL-1β in the culture supernatant of mouse BMNs primed with LPS (0.25 μg/mL) followed by ATP (2.5 mM) treatment in the presence of ac-YVAD-cmk (20 μM) (n = 3). (**C**) Quantification of IL-1β in the culture supernatant of *Nlrp3* $^{+/+}$ or *Nlrp3* $^{-/-}$ mouse BMNs treated with LPS (0.25 μg/mL) followed by ATP (2.5 mM) or nigericin (5 μM) treatment. (n = 3) (**D**) Quantification of IL-1β in the culture supernatant (upper panel) and immunoblots (lower panel) of mouse BMNs or BMDMs primed with LPS (0.25 μg/mL) followed by ATP treatment (2.5 mM) (n = 3). Culture supernatants (Sup) or cellular lysates (Lys) were immunoblotted with the indicated antibodies.

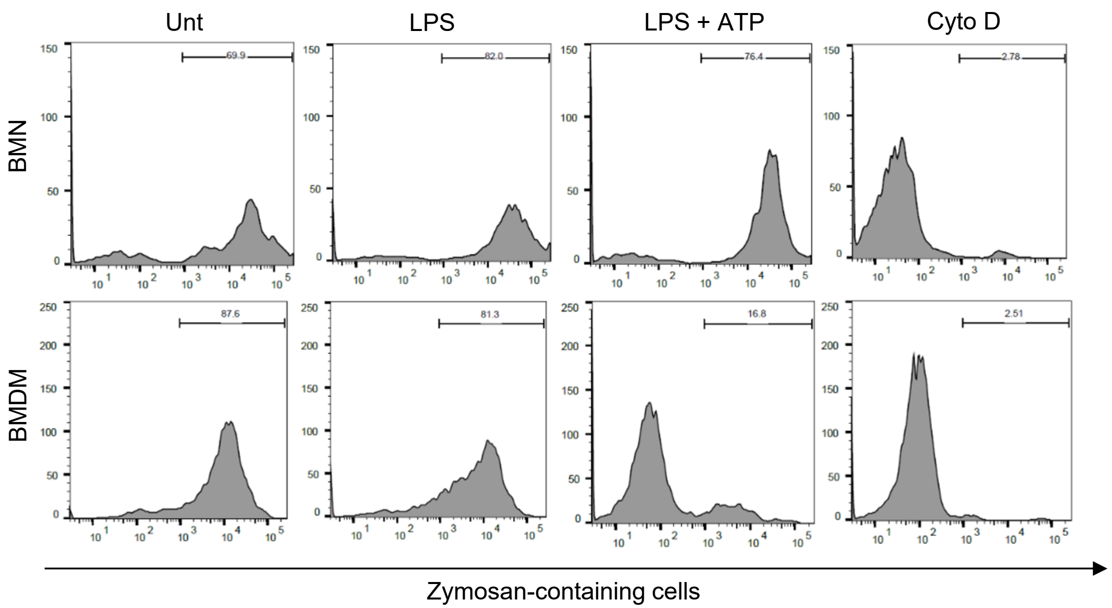

Supplementary Figure S10. Phagocytic activity of neutrophils and macrophages after inflammasome activation. Mouse BMNs or BMDMs were untreated (Unt) or treated with LPS (0.25 μg/mL, 2.5 h) alone, LPS followed by ATP (2.5 mM, 1 h), or cytochalasin D (Cyto D, 10 μM, 30 min) as determined using flow cytometric analysis after incubating with zymosan-FITC (5 particles/cell, 30 min)

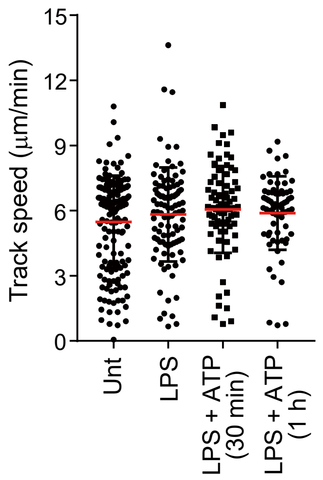

Supplementary Figure S11. Migration ability of inflammasome-active neutrophils. Quantification of track speed of BMNs treated with LPS (0.25 μg/mL, 2.5 h) alone or followed by ATP (2.5 mM), as determined using *in vitro* migration assays.

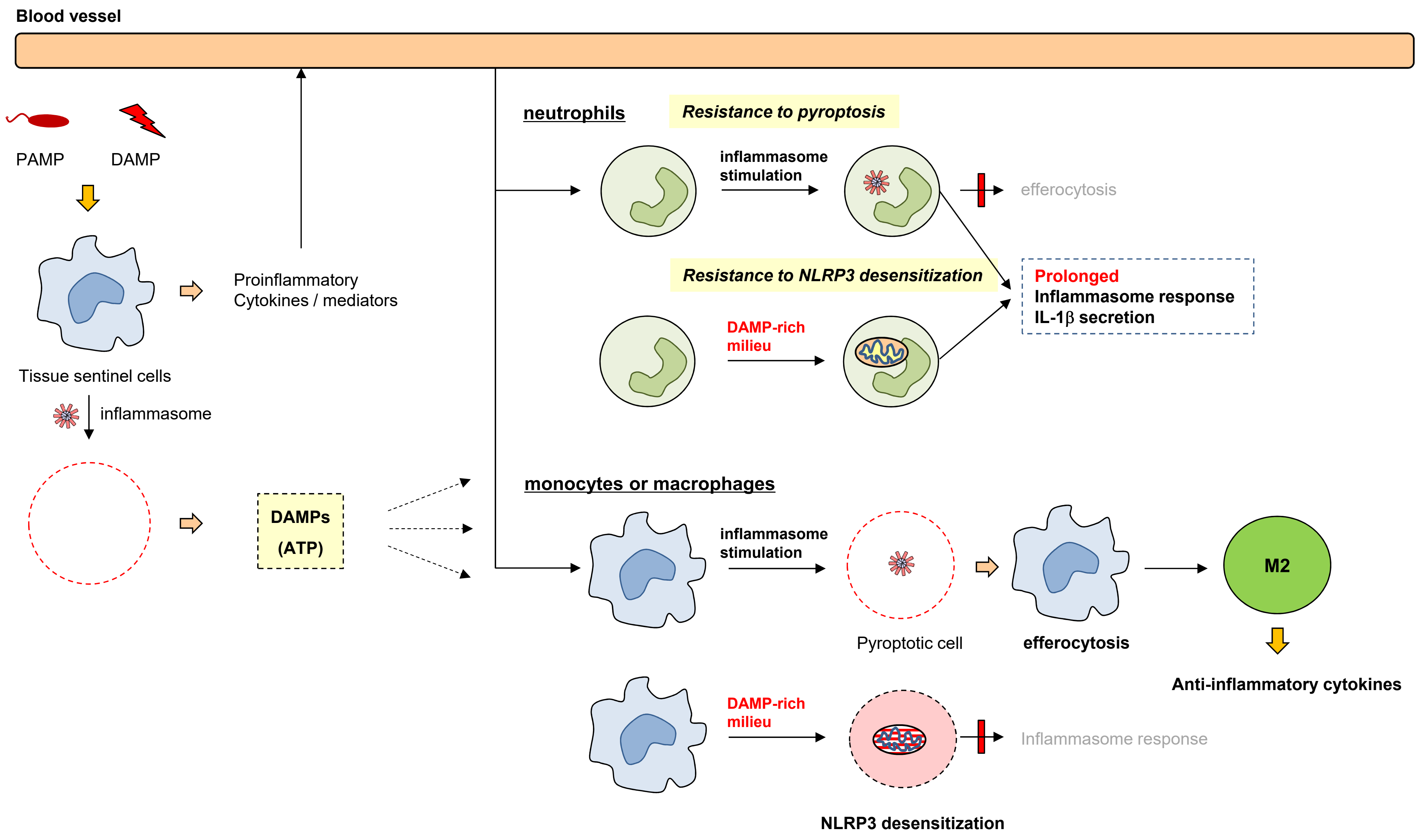

Supplementary Figure S12. Proposed role of neutrophils for inflammasome-mediated responses in the DAMP-rich milieu.
